# Current Challenges in Understanding the Cellular and Molecular Mechanisms in Niemann–Pick Disease Type C1

**DOI:** 10.3390/ijms20184392

**Published:** 2019-09-06

**Authors:** Anja U. Bräuer, Angela Kuhla, Carsten Holzmann, Andreas Wree, Martin Witt

**Affiliations:** 1 Research Group Anatomy, School of Medicine and Health Sciences, Carl von Ossietzky University Oldenburg, D-26129 Oldenburg, Germany; 2 Research Center for Neurosensory Science, Carl von Ossietzky University Oldenburg, D-26129 Oldenburg, Germany; 3 Institute for Experimental Surgery, Rostock University Medical Center, Schillingallee 69a, 18057 Rostock, Germany; 4 Center of Transdisciplinary Neuroscience Rostock, D-18147 Rostock, Germany (C.H.) (A.W.); 5 Institute of Medical Genetics, Rostock University Medical Center, D-18057 Rostock, Germany; 6 Institute of Anatomy, Rostock University Medical Center, D-18057 Rostock, Germany

**Keywords:** neurodegeneration, lipid storage disorder, RT-PCR, histology, electron microscopy, cholesterol homeostasis, miglustat, cyclodextrin, treatment

## Abstract

Rare diseases are a heterogeneous group of very different clinical syndromes. Their most common causes are defects in the hereditary material, and they can therefore be passed on to descendants. Rare diseases become manifest in almost all organs and often have a systemic expressivity, i.e., they affect several organs simultaneously. An effective causal therapy is often not available and can only be developed when the underlying causes of the disease are understood. In this review, we focus on Niemann–Pick disease type C1 (NPC1), which is a rare lipid-storage disorder. Lipids, in particular phospholipids, are a major component of the cell membrane and play important roles in cellular functions, such as extracellular receptor signaling, intracellular second messengers and cellular pressure regulation. An excessive storage of fats, as seen in NPC1, can cause permanent damage to cells and tissues in the brain and peripheral nervous system, but also in other parts of the body. Here, we summarize the impact of NPC1 pathology on several organ systems, as revealed in experimental animal models and humans, and give an overview of current available treatment options.

## 1. Introduction

The rare Niemann–Pick disease type C1 (NPC1) is an autosomal–recessive, lipid-storage disorder characterized by neonatal jaundice, hepatosplenomegaly, and progressive neurodegeneration [1,2,3]. The mutation responsible for approximately 95% of these cases has been mapped to a gene on chromosome 18q11 designated *NPC1* [4]. The progressive neurodegeneration induces ataxia, dystonia, and impairment of intellectual function [2,5,6]. The NPC1 protein is involved in intracellular lipid trafficking [7,8]. The defect caused by mutations in the *NPC1* gene induces accumulation of unesterified cholesterol, glycosphingolipids, and other fatty acids in the endosomal/lysosomal system [9]. This impaired lipid transport leads particularly to an extensive loss of Purkinje cells in the cerebellum and degeneration of other central nervous compartments [10,11,12,13]. Although all NPC1 cells show cholesterol and glycosphingolipid accumulation, the major clinical impact is in the liver and brain [14]. Many affected individuals have liver disease at birth. Most begin to show symptoms of neurodegeneration as young children, with learning difficulties and motor coordination problems being paramount. These individuals typically die in their teen years. There is also an infantile form of the disease; these infants show hepatosplenomegaly, fail to thrive, and die within 2–3 years. Other individuals with a milder form of the disease enjoy a normal childhood and are diagnosed as adults, with early dementia as the predominant symptom. NPC was little studied in the past because of its rarity; NPC1 is diagnosed in one in every 92,000–150,000 births, though recent genome and exome analysis including late appearing phenotypes predicts an increased incidence of one in 20,000–39,000 births [15]. However, biomedical scientists took more notice when the *NPC1* gene was shown to encode a large membrane protein with features shared by several key regulators of cholesterol homeostasis. Identification of the NPC2 protein by Lobel′s laboratory in 2000 [1] revealed a small soluble glycoprotein that likely partners with NPC1 in transporting lipids.

## 2. Lipid Trafficking and NPC1 (Niemann–Pick Disease Type C1)

### 2.1. Cholesterol Transport

Cholesterol homeostasis is essential for the functional integrity of the cell [3]. Nearly all cells in the body, including neurons of the central nervous system (CNS), take up cholesteryl ester and/or unesterified cholesterol carried in various lipoproteins from the surrounding pericellular fluid by receptor-mediated and bulk-phase endocytosis [16,17]. Both the cellular content and distribution of cholesterol within the cell are highly dynamic and tightly regulated through de novo synthesis of cholesterol by the endoplasmic reticulum (ER) [4], and by uptake of cholesterol ester-rich lipoprotein particles circulating in the serum by the low-density lipoprotein (LDL) receptor pathway [9]. The main sorting station for cholesterol within the cell is the late endosome (LE), an intermediate stage in the endosomal–lysosomal trafficking pathway. Two LE proteins, NPC1 and NPC2, appear to be key players that initiate the sorting process [18,19,20,21].

### 2.2. NPC Protein Function

#### 2.2.1. NPC1

NPC1 is a large glycoprotein with 13 transmembrane-spanning domains that is found in LE [22]. It contains a five-transmembrane domain, called the ‘sterol-sensing domain’ that it is found in multiple other proteins hypothesized to sense the cholesterol content of their surroundings. A large hydrophilic N-terminal domain and two hydrophilic loops extend into the endosome lumen, but functions and/or binding partners are unknown. In the steady state, most NPC1 protein is found in LE, but the protein is present in tubules and vesicles that bud off from endosomes, traffic across the cell and then return [23]. The physiological importance of the NPC1 protein is emphasized by its conservation (yeast, insects, worms, and mammals all have NPC1), although these organisms diverge considerably in their need for, and handling of, sterols.

#### 2.2.2. NPC2

**Figure 1:**
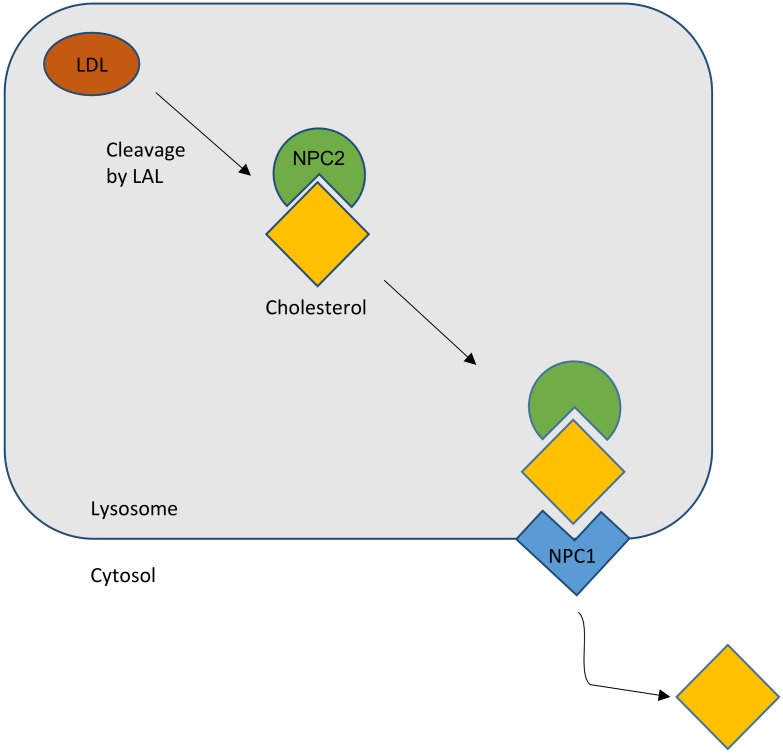
Schematic outline of the normal cholesterol trafficking via NPC2 and NPC1 interaction. LAL, lysosomal acid lipase (courtesy of René Thiemer, modified after [26]).

The *NPC2* gene encodes a protein with the reassuring features of a bona fide lipid-transport protein. NPC2 is a soluble glycoprotein that is delivered to lysosomes by virtue of its mannose phosphate moiety [1]. It is also secreted and found in epididymal fluid, bile, and milk. The secreted protein was purified in apo- and sterol-bound forms [24]. Apo-NPC2 was found to have an incipient ligand-binding pocket, which expands to accommodate cholesterol (Figure 1). NPC2 was shown, in in vitro assays, to rapidly transport cholesterol from donor to acceptor membranes via a collisional mechanism [25]. As might be expected for a lysosomal protein, transfer activity was greater in an acidic environment and was enhanced by the presence of the late-endosome/lysosome (LE/LY) -specific lipid lysobisphosphatidic acid.

In summary, the most favored hypothesis is that, as a lipid cargo is brought to the LE/LY, the lipids are digested into their constituent molecules. NPC2 facilitates the transfer of cholesterol, and perhaps other lipids, to the delimiting membrane of the organelle. NPC1 senses the rising membrane cholesterol content and signals for the membrane to bud, carrying cargo to destinations throughout the cell (Figure 1).

### 2.3. Diagnostic Tools

**Figure 2:**
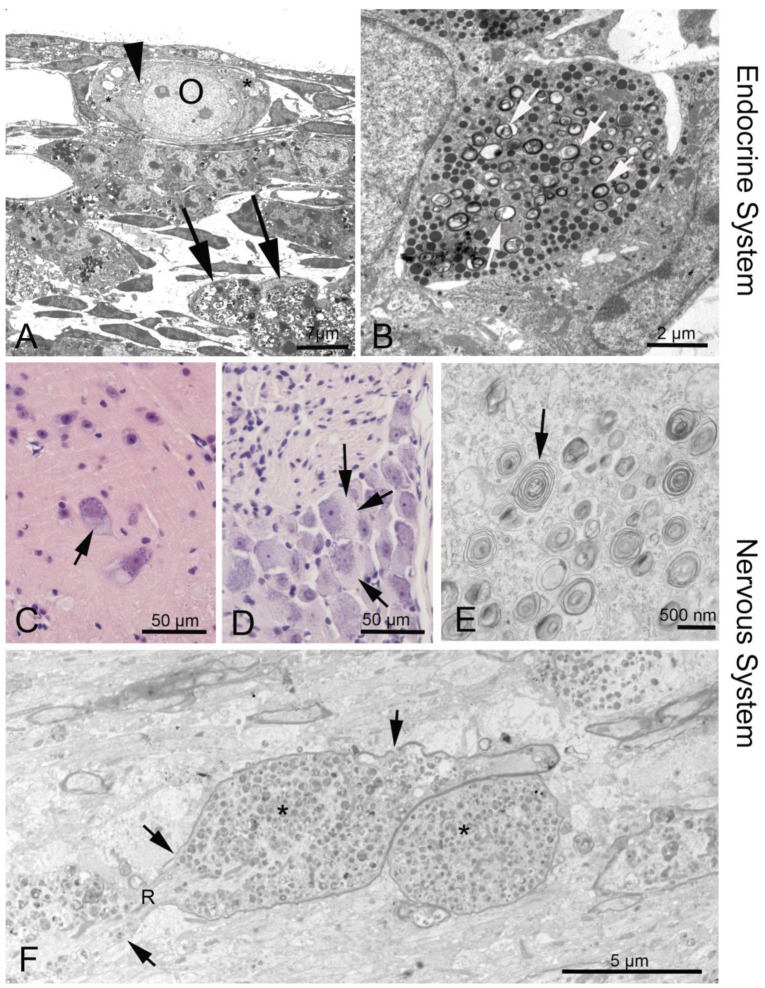
Phenotypes of NPC1 in the endocrine and nervous system. (**A**) Ovary of an *Npc1^−/−^* mouse. The oocyte (O) contains enlarged endoplasmic reticulum (ER) with myelin-like deposits (arrowhead), as does the surrounding follicular epithelial cell (asterisk). Large accumulations are seen in a macrophage (arrows). (**B**) Neuroendocrine cell in the anterior pituitary. Arrows point at myelin-like inclusions between secretory vesicles. (**C**) Some alpha motor neurons in the anterior horn of the spinal cord are filled with light material replacing the darker perinuclear Nissl substance of the endoplasmic reticulum. (**D**) Similar damage is seen in dorsal root ganglion cells (arrows). (**F**) Corpus callosum: A longitudinal nerve fiber is enlarged and congested by autophagosome content (asterisks) that interrupts the continuity of neurofilaments and neurotubuli. The myelin sheath (arrows) has also thinned and disintegrated. R, node of Ranvier.

The distinct heterogeneity of this disease makes it difficult to diagnose. There are several options to diagnose NPC: skin and liver biopsy for filipin staining of cultured fibroblasts; electron microscopic analysis of vacuolation or hepatocytes containing “myelin figures” (Figure 2) [27,28,29]; molecular genetic analysis with direct sequencing of *NPC1* and *NPC2* gene mutations [28]; bone marrow aspiration for the detection of foamy histiocytes [30,31]; and use of cholesterol esterification assays and oxysterol assay-based screening to measure the increase of cholestane-3β,5α,6β-triol (cholesterol oxidation product, “triol”) [32,33,34,35]. A possible non-chemical biomarker and treatment control may consist in olfactory testing, since olfactory deficits may mirror the progress of the disease [36]. However, as yet there are no human data available [27].

### 2.4. Therapies

So far, there is no causal therapy of NPC1, though the iminosugar miglustat (Zavesca^®^) is the only approved drug in Europe used for supporting and symptomatic therapy in NPC1 [37]. Miglustat is a small molecule that inhibits glycosylceramid synthase, one of the key components of the glycosphingolipid biosynthesis, therefore reducing intracellular lipid storage [38]. Long-term therapy with miglustat has been shown to increase lifespan and stabilize neurological functions. Additionally, miglustat has been ascribed activity against oxidative stress [39]. However, limitations consist in mainly gastrointestinal side effects such as diarrhea, weight decrease, and flatulence, but also tremor [40]. A further promising drug, 2-hydroxypropyl-β-cyclodextrin (HPβCD)—a cyclic oligosaccharide—is used as an enabling excipient in pharmaceutical formulations, as well as a cholesterol modifier in vivo. Therapy results in delayed onset of neurological symptoms with increased lifespan [37,38,41]. Matsuo et al. [42] reported in a clinical trial that HPβCD was effective in NPC1 patients, suggesting that HPβCD is a promising drug candidate in NPC1 disease. HPβCD overcomes the transport defect leading to excretion of accumulated cholesterol as bile acid, as shown in *Npc1^−/−^* mice [43]. It has been suggested that cholesterol efflux is mediated by the ATP binding cassette subfamily G member 1 (ABCG1), which promotes biliary excretion of sterols, ameliorating liver function [43,44]. Unfortunately, HPβCD administration also has side effects, particularly on the survival of outer hair cells, leading to hearing loss. This major side effect occurs in a dose- and duration-dependent manner [45,46]. What is more, in an open-label, dose-escalation phase 1–2a study, promising effects of HPβCD were recorded [47]; however, preliminary results of a current multinational phase 2b/3 clinical study involving about 50 patients treated with 200 mg/kg intrathecally applied HPβCD every 2 weeks indicate doubts that HPβCD achieves benefits when compared to a placebo [48,49,50].

Another promising therapy, so far applied only in animal models, consists of a combination of miglustat, the neurosteroid allopregnanolone, and HPβCD [13,51,52,53], resulting in further prevention of cerebellar Purkinje cell loss, improved motor function, reduced intracellular lipid storage, and prolonged life span in *Npc1^−/−^* mice. 

Another therapeutic approach showed that the activity of the liver X receptor β (LXR β) can regulate the cholesterol flux from the brain, which leads to a reduction of neuroinflammation and slows therefore the neurodegeneration process. However, these positive effects result only in a modest lifespan prolongation [54,55]. Nevertheless, LXR β activation by treatment with an LXR agonist (T1317) can be useful in combination, e. g., with HPβCD. 

In the absence of a causal treatment, there is still, a need to identify novel treatment strategies. Currently, histone deacetylase inhibitors (HDACi) are a focus of interest, due to the findings that they can reduce cholesterol accumulation in LE/LY [55,56,57,58]. These enzymes mediate posttranslational deacetylation of many types of proteins, e.g., histones, transcription factors, and chaperones [59]. In spite of its interaction with many different proteins and signaling pathways, it has been shown that HDACi increases expression of the low-activity mutant NPC1 protein [56,57], at least in vitro. 

A further treatment option is FTY720 (fingolimod), a sphingosin analog. This drug is already approved for human use to treat multiple sclerosis [60]. FTY720 can enter the cell nucleus, where it is phosphorylated by sphingosine kinase 2 (SphK2). This active form is an inhibitor of class I histone deacetylases. The advantage of this drug over available HDACi is to regulate the expression of only a limited number of genes, which are restricted to cholesterol and sphingolipid metabolism, compared with the large number (thousands of genes [61]), which are activated by HDACi [60].

Another treatment approach is the application of arimoclomol, a coinducer of heat shock protein 70 (HSP 70) that improves the binding of several sphingolipid-degrading enzymes to their essential cofactor bis(monoacyl)glycerophosphate in vitro [62,63]. Beneficial effects for NPC patients have also been observed with drugs such as ursodeoxycholic acid [64,65] and acetyl-DL-leucine [66].

Moreover, an increased level of functional NPC1 can be achieved using gene therapy [67]. In some studies, it has been shown that the adeno-associated virus (AAV) 9 vector may successfully transfer the *NPC1* gene into the CNS of *Npc1^−/−^* mice [67,68,69]. Systemic delivery of a functional *NPC1* gene into *Npc1^−/−^* mice significantly extends the lifespan, ameliorates neurodegeneration, and improves behavioral abnormalities [67,68,69]. The current state of this research is promising.

## 3. Pathology of NPC1 in Humans and Mice

In the following, we give a systematic overview of NPC1 pathology in various, mostly peripheral, tissues and organs. We also compile our own behavioral and novel histopathological material using the Jackson mouse strain BALB/*cNctr-Npc1* ^m1N/−^J (see Supplementary Materials). 

### 3.1. Behavior

A combined therapy using HPβCD, allopregnanolone, and miglustat has been shown to delay disease onset and increase the lifespan of *Npc1* mutant mice by reducing intraneuronal lipid storage [51]. Encouraged by these findings, the effects of therapeutic drugs on the behavior of *Npc1^−/−^* mice (BALB/cJ NPC1NIH) were evaluated in several studies. The effects of HPβCD/ allopregnanolone/miglustat combination therapy on motor and cognitive performance of *Npc1* mutant mice was first explored using standard behavioral tests [53]. Combination-treatment of mutant mice significantly and positively influenced motor dysfunction in an open field and elevated plus maze and accelerod tests when compared to sham-treated mutant mice. Spatial learning in the Morris water maze, however, did not benefit from therapy [53].

A follow-up study addressed the question of possible side effects of therapeutic drugs [70]. For this purpose, a battery of standard behavioral tests was used on healthy, wild-type mice to study pharmacological effects of miglustat as a monotherapy, in comparison to the well-known combination therapy [51,53]. Combination treatment caused reduced brain and body weights, whereas miglustat alone led to reduced brain weight but unaltered body weight. Motor capabilities and spontaneous motor behavior were unaltered in both drug-treated groups. However, miglustat-treated mice displayed impaired spatial learning compared to sham- and combination-treated wild-type mice. Both combination- and miglustat-treated mice showed enhanced anxiety in the elevated plus maze compared to sham-treated mice. The authors suggested that HPβCD/allopregnanolone ameliorates most side effects of miglustat in wild-type mice [70]. However, further behavioral studies are needed to investigate the effects of each drug separately.

Another therapy strategy used miglustat in combination with the calcium modulator curcumin and the anti-inflammatory drug ibuprofen in *Npc1^−/−^* mice [71]. Motor function and coordination was evaluated by measuring rearing ability in an open-field test. The positive effect of miglustat monotherapy was further improved by additional dual therapy with curcumin and miglustat, and the triple combination therapy [71]. However, the authors have not performed behavioral tests for cognition and anxiety. 

### 3.2. Sensory Systems

#### 3.2.1. Hearing 

In contrast to general neurological deficits, sensory systems in NPC1 have attracted only minor diagnostic and research attention. Only a few studies refer to auditory phenotypes in NPC1 in patients [72] and in mice [73], although the most important approach to the auditory system came with the observation that HPβCD as an effective treatment agent (see above) can lead to severe hearing loss [45,52,74]. Outer hair cells seem to be the most susceptible targets of CD [45,75], but the reasons for the ototoxicity are currently not clear [52]. Pathological brainstem recordings also indicate auditory pathway involvement in NPC1 patients [76].

#### 3.2.2. Vision

Visual deficits were first published by Claudepierre et al. [77] in *Npc1* mutant mice and showed that lack of Npc1 leads to pathological electroretinogram responses, retinal degeneration with disruption of the retinal pigment epithelium, and degeneration of bipolar and optic ganglion cells. NPC1-typical autophagosomes were also found in glia and nerve fibers along the optic nerve in mutant mice [78]. Functionally, *Npc1* pathology in mice leads to degenerated visual pathways, as revealed by analyzing visual evoked potentials [79]. ERG recordings of NPC1 patients have not yet been performed, but saccadic eye movements have been often observed in adult patients, reflecting general neurological deficits at the levels of frontal eye fields and the brainstem [80], but there are no reports on retinal changes in patients who underwent treatment. 

#### 3.2.3. Olfaction

As observed in several other neurodegenerative disorders, the *Npc1* mutation also elicits olfactory deficits, at least in experimental animals [81]. Peripheral olfactory receptor neurons and associated supporting glia are severely damaged, leading to hyposmia in *Npc1^−/−^* mice [81], which goes along with reduced electrophysiological responses in the olfactory mucosa of older animals [81]. Considerable damage to olfactory receptor cells is accompanied by a downregulation of certain, but not all, olfactory receptors [36], and is accompanied by severe central astro- and microgliosis of the olfactory bulb [81,82]. Due to the extraordinary plasticity of the olfactory system, regenerative proliferation activities have been observed both in the olfactory mucosa [83] and at the CNS level [36,82]. All these effects could be prevented by treatments with HPβCD (see above). Olfactory performance in humans has not yet been investigated. 

#### 3.2.4. Peripheral Nervous System

As most tissues are affected in NPC1, and especially CNS disorders play a crucial role (Figure 2E), it should not be surprising that peripheral nerves are also involved. However, there are few systematic reports on the peripheral nervous system, and no documentation on treatment. Bagel et al. [84] report myelination defects of Schwann cells, rather than axonal damage, in cats. Electrophysiological studies showed altered lower-limb somatosensory evoked potentials in adult patients [76].

Our group noted neuronal cell degeneration in dorsal root ganglia (Figure 2C,D) as well as in satellite cells of the trigeminal nerve, and also described deposits within trigeminal ganglion cells in *Npc1^−/−^* mice. Similar results were obtained earlier in the acid sphingomyelinase knock-out (ASMKO) mouse model of Niemann–Pick disease type A [85].

### 3.3. Endocrine Organs and Reproductive System

Endocrine disorders are not the focus of research on human NPC1 mutations, and the few available data refer mostly to *Npc1* mouse models. Female *Npc1^−/−^* mice are infertile, probably by failure of the hypothalamic control of the pituitary [86]. Gévry et al. described hypoplastic pituitaries with prolactin expression. Failing ovulation and missing corpora lutea in the *Npc1^−/−^* mutant mouse could be overcome with gonadotropin treatment [87]. Our electron microscopic studies demonstrate discrete myelin-like deposits in diverse hormone-producing cells of the anterior pituitary and similar inclusions in follicular epithelial cells of the ovary (Figure 2A,B). Accordingly, cholesterol balance is also disturbed in male *Npc1^−/−^* mice [88], leading to sperm defects and altered testosterone production [89,90].

In the adrenal gland, Elleder and Smid [91] observed only mild morphological changes, mainly in stromal cells, but barely in the cortex, and not at all in the medulla. This is confirmed by our own histological studies in *Npc1^−/−^* mice (not shown). 

### 3.4. Gastrointestinal Tract (GI)

**Figure 3:**
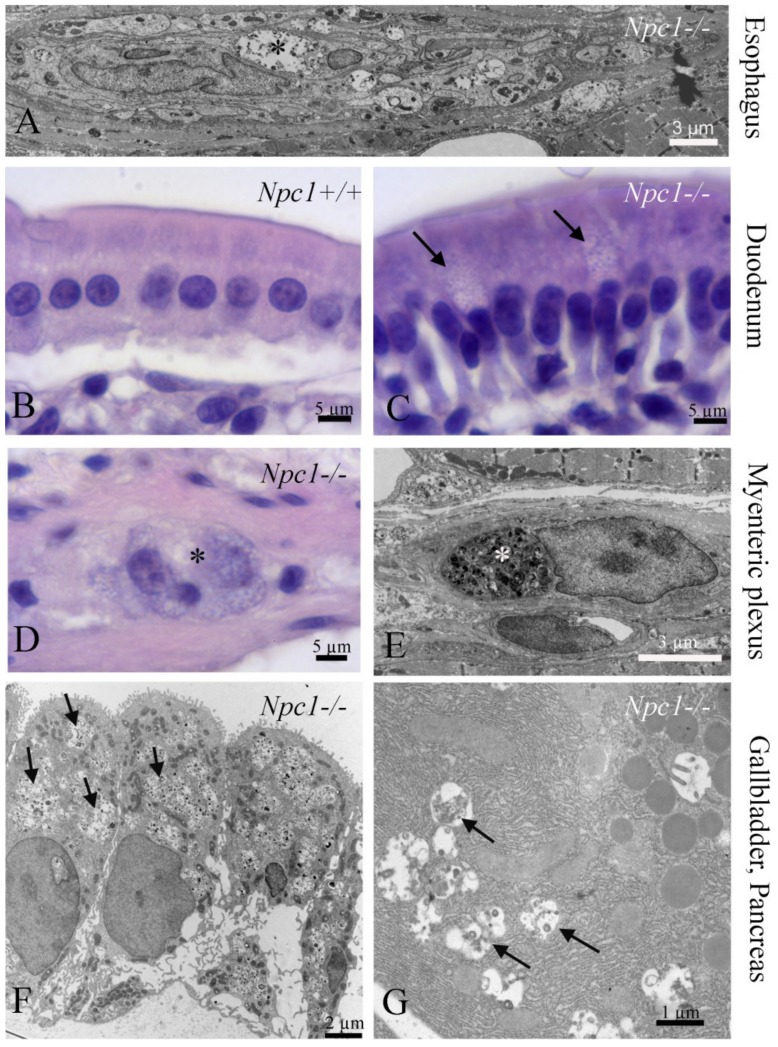
Phenotypes of NPC1 in the gastrointestinal tract. (**A**) Myenteric plexus in the esophagus with myelin-like inclusions in glia cells (asterisk). (**B**) Normal duodenal enterocytes in *Npc1^+/+^*, and (**C**) pathological cells (arrows) in an *Npc1^−/−^* mouse. (**D**,**E**) Autophagosomes in ganglion cells of the myenteric plexus in the duodenum. (**F**) LE/LY storage in epithelium of the gallbladder and (**G**), in acinar cells of the exocrine pancreas.

The GI tract has been focused as the “visceral type” of NPC1, especially the largest associated gland, the liver. Apart from the lipogenetic potential of the liver, we observed a plethora of cell types and tissues in the GI tract that contain NPC1-typical deposits, e.g., epithelial cells of the duodenal mucosa (Figure 3B,C) and the gallbladder (Figure 3F), as well as within the ganglionic plexuses (Figure 3A,D,E). This may be associated with the delayed intestinal transit [92]. In the same *Npc1^−/−^* mouse model, Cougnoux et al. [93] observed significant increases in cyanobacteria and epsilon-proteobacteria, as well as macrophage dysfunction, within the GI tract. Additionally, a high frequency of Crohn’s disease has been observed in NPC1 patients [94,95,96], though the pathomechanisms of Crohn′s disease in NPC1 are different with regard to microbiota changes, at least in the widely used *Npc1* mouse model [93].

### 3.5. Pancreas

The NPC1 condition does not seem to produce clinically important effects in both endocrine and exocrine pancreas; there is only one report on morphological changes in acinar cells in NPC [97]. We observed only some myelin-like inclusions in pancreatic acinar cells, but no major structural damage (Figure 3G).

### 3.6. Lung

**Figure 4:**
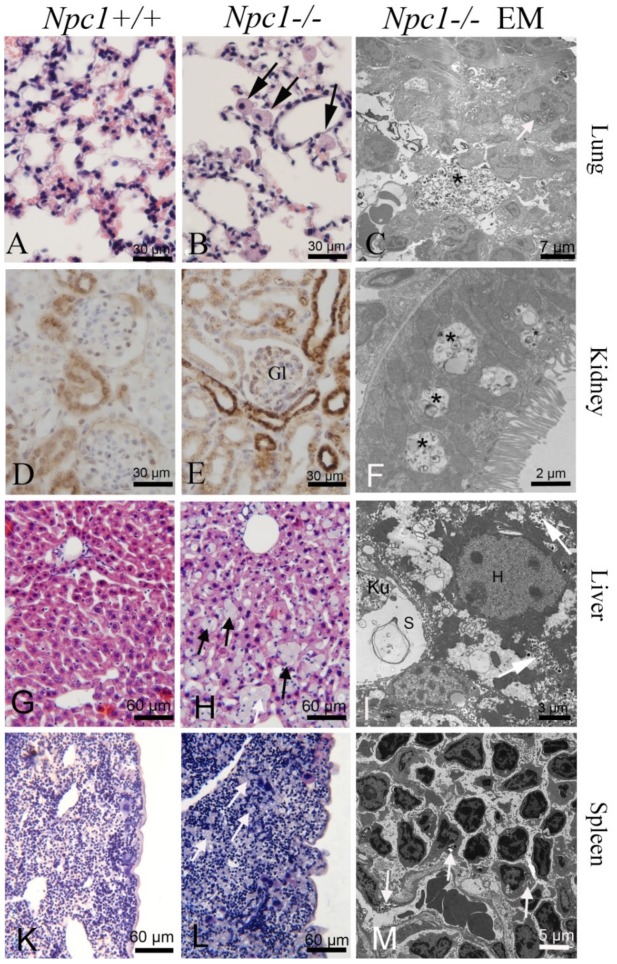
Phenotypes of NPC1 in visceral organs. Left column (**A,D,G,K**): wildtype control animals (*Npc1^+/+^),* middle column: mutant mice (*Npc1^−/−^*), right column: mutant animals at the electron microscopic level. The lung presents alveolar edema and emphysema (**B**) and many activated pulmonary macrophages (foam cells, arrows). (**C**) Detailed view of a macrophage filled up with autophagosomes (asterisk) and a normal alveolar cell type II (arrow). In the kidney, cathepsin D immunoreactivity shows increased lysosomal activity in *Npc1^−/−^* mice (**D**), especially in proximal tubules and endothelial cells, as well as podocytes of the glomerulus (**E**, Gl). (**F**) Myelin-like deposits (asterisks) in proximal tubular cells. In the liver, numerous pale cells are also visible (**H**, arrows). (**I**) Kupffer cells (Ku) in the sinusoids (S) and hepatocytes (H) are filled with autophagosomes (arrows). (**L**) In the spleen, numerous foam cells are seen (arrows). (**M**) Inclusions in endothelial cells and macrophages (arrows).

Although NPC1 affects almost all tissues, pulmonary dysfunction has rarely been shown in NPC1 patients compared to NPC2. It is, however, more common in NPC1 patients than previously known [98]. Recently, Roszell et al. [99] found severe pathological structural alterations in all cell types of the blood–air barrier in NPC1 mouse and cat models, and mainly altered surfactant-producing type II cells, elevated levels of phospholipids in the alveolar space, larger and more numerous macrophages, and thickening of the alveolar septum. In the Npc^nmf164^ model, Erickson et al. [100] found even aggravated pulmonary pathology after nasal inhalation of HPβCD. Histological features of murine NPC1-caused lung disease are shown in Figure 4A–C. Many patients suffer from incurable bacterial or viral bronchopulmonary infections that are exacerbated by NPC1-predamaged pulmonary tissue [101].

### 3.7. Kidney

In *Npc1* mutant mice, cholesterol deposits are increased in the kidney [43]. Lipid inclusions are observed in all parts of the tubular system, especially the proximal tubules, but also podocytes and endothelium in the glomeruli are affected (Figure 4D–F). Similar observations have been reported only in a related mouse model mimicking human NPC type A [102]. The kidney, however, plays an important part during therapy with HPβCD, as this agent is completely cleared from the plasma by the kidney [43,103]. 

### 3.8. Liver and Biliary Tract

Liver plays the key role in the clearance of circulating cholesterol carried in lipoproteins. In both human and mouse with a mutation in *NPC1*, the liver represents the highest rate of sterol accumulation, which forms a basis for the development of liver disease. Aside from hepatomegaly, Niemann–Pick patients often suffer from prolonged neonatal jaundice and ascites, as well as liver failure [104,105,106,107]. Furthermore, it is known that the *NPC1* mutation is the second-most common cause of neonatal cholestasis [108], whereby 10% of these patients die due to liver failure [29]. Accordingly, livers of *Npc1^−/−^* mutant mice reveal enhanced liver-tissue damage and inflammation indicated by raised hepatic apoptosis, as well as necrosis and infiltration of foamy macrophages and increased proliferation of hepatic stellate cells, resulting in liver fibrosis (Figure 4G–I) [44,109,110,111]. Moreover, the biliary tract is of some importance in *Npc1^−/−^*, since bile acid metabolites such as plasma oxysterols, cholestan-3β,5α,6β-triol (Triol), and 7-ketocholesterol (7-KC) play an increasing role as biomarkers for NPC1. 

The cause for all these pathological hallmarks is, beside the *NPC1* mutation-associated cholesterol accumulation, the concomitant dysregulation of hepatic lipid metabolism. Cholesterol homeostasis is characterized by a balance of lipogenesis and lipolysis. Lipogenesis is mainly regulated by the nuclear receptor liver X receptor (lxr) and its target genes, including, among others, sterol regulatory element-binding transcription factor 1c (srebp1c), which is known to be upregulated in livers of *Npc1^−/−^* mutant mice [43]. Beside the sterol regulatory element binding protein (SREBP) pathway [112], the peroxisome proliferator-activated receptor (PPAR) pathway with the genes peroxisomal acyl-coenzyme A oxidase 1 (acox1) and fatty acid transport protein 2 (fatp2) is implicated in the regulation of free fatty acid hepatic metabolism, which was found downregulated in *Npc1^−/−^* mutant mice [44]. Moreover, the peroxisome proliferator-activated receptor α (pparα) regulates the expression of liver genes involved in mitochondrial and/or peroxisomal fatty acid β-oxidation, meaning an enhanced lipolysis. In several studies, it was reported that pparα gene expression is decreased in livers of *Npc1^−/−^* mutant mice [44,112]. Therefore, decreased expression of the pparα gene provides an additional plausible explanation for the accumulation of free fatty acids in NPC1 disease. Target downregulated genes of pparα in *Npc1^−/−^* mutant mice are apolipoprotein E (apoE) and ATP-binding cassette A1 (abca1) [44], which are involved in transporting cholesterol to the plasma membrane [113]. In summary, the observed shift of the lipid metabolism towards lipogenesis with simultaneously reduced lipolysis most probably supports hepatic steatosis and underlines the pathological relevance of nuclear receptors in both human and mouse with an *Npc1* mutation. The histological phenotype of NPC1-affected liver tissue is displayed in Figure 1G–I. Therapeutic approaches showed that both the combination therapy and HPβCD monotherapy ameliorate NPC1 liver disease symptoms by causing a reduction of hepatic lipids. In this context, the therapeutic effect is related to pparα- and acox1-associated lipolysis/β-oxidation and to fatp2-induced fatty acid transport [31,32,114].

### 3.9. Spleen and Lymphatic System

Data on a disturbed balance within splenocyte subpopulations in humans is currently not available. As a matter of fact, irregular consequences of NPC1 malfunction with regard to the immune system have not yet been described in NPC1, in contrast to other storage diseases. For example, increased autoantibody formation has been found in Gaucher and Fabry´s disease (reviewed by [115]). The histological features of *Npc1^−/−^* affected mouse spleen is shown in Figure 4K–M. There is apparent infiltration with spleen macrophages (foam cells). A recent fluorescence activated cell sorting (FACS) analysis revealed that increased numbers of splenic myeloid cells in *Npc1^−/−^* mice were normalized by a combination treatment with miglustat and HPβCD. Treated *Npc1^−/−^* mice also showed decreased numbers of cytotoxic T cells and increased numbers of T helper cells [116].

### 3.10. Cardiovascular System

**Figure 5:**
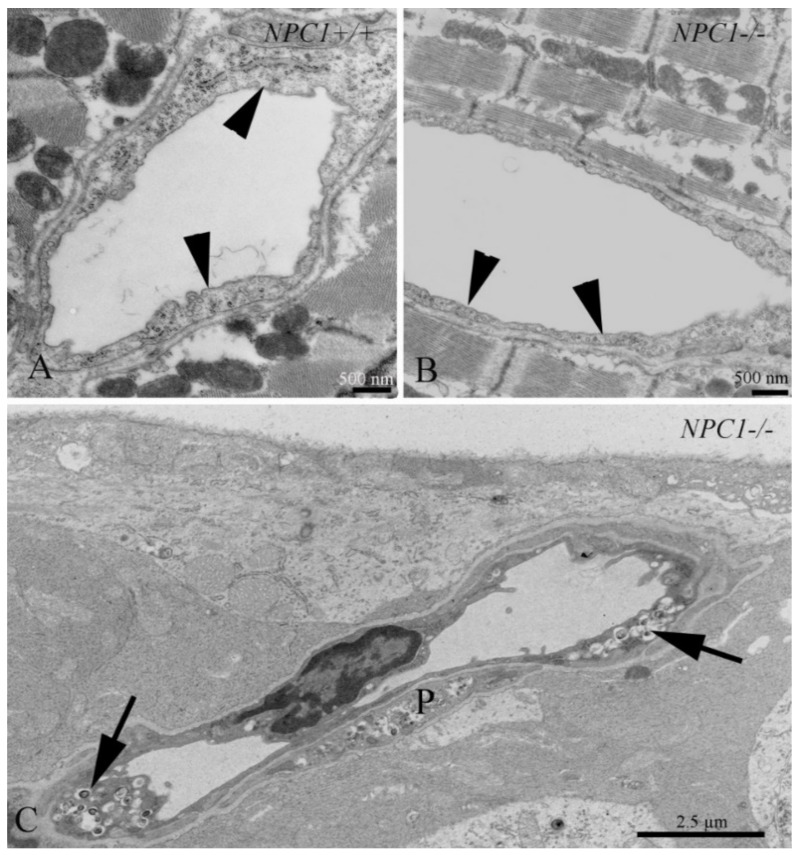
Phenotypes of NPC1 in the cardiovascular system. (**A**) Cardiac muscle with an endothelial cell (arrowheads) in an *Npc1^+/+^* (control) mouse. (**B**) Endothelial cells, in contrast to most other organs, do not contain lipid-like inclusions. (**C**) Capillary (arrows) and a pericyte (P) in the ganglion cell layer of the retina, are filled with NPC1-typical autophagosomes.

It is well known that the formation of foam cells or atherosclerotic plaque is the hallmark event leading to coronary heart disease [117,118]. The endothelium in many organ systems in NPC1 is typically so affected (Figure 5), and cholesterol imbalance may lead to clinically adverse situations such as arteriosclerosis. Foam cells in atherosclerotic lesions derive from macrophages and vascular smooth muscle cells [119]. Recent reports have shown that atherogenesis may be prevented by HPβCD [120,121,122]. Interestingly, cardiac muscle and associated tissues are almost free of NPC1-related pathology (Figure 5) and do not as yet play a major role in diagnostic and therapeutic considerations of NPC1.

### 3.11. Tooth

Caries risk and activity can be a problem in patients, not only with NPC, but also with other neurological or psychiatric diseases, particularly affecting children. Disabled children often do not chew and the daily oral hygiene activities are reduced [123]. 

## 4. Perspectives

Until now, over 6000 rare diseases have been characterized by a broad diversity of disorders and symptoms that vary not only from disease to disease, but also from patient to patient suffering from the same disease. There is a strong need to develop novel treatments and understanding of how the therapies work. From lipid-storage diseases, such as NPC1, we can learn more about the cellular and molecular mechanisms underlying neurodegeneration. We should, therefore, give these rare diseases more attention.

## Supplementary Materials

Supplementary materials can be found at https://www.mdpi.com/1422-0067/20/18/4392/s1.

## Abbreviations

AAV	adeno-associated virus
ABC	ATP binding cassette
ACMKO	acid sphingomyelinase knock-out
acox1	acyl-coenzyme A oxidase 1
apoE	apolipoprotein E
ASMKO	acid sphingomyelinase knock-out
CNS	central nervous system
ER	endoplasmic reticulum
FACS	fluorescence activated cell sorting
fatp2	fatty acid transport protein 2
GI	gastrointestinal
HDACi	histone deacetylase inhibitors
HPβCD	2-hydroxypropyl-β-cyclodextrin
LDL	low-density lipoprotein
LE/LY	late endosome/lysosome
lxr	liver X receptor
NPC1	Niemann–Pick disease type C1
PPAR	peroxisome proliferator-activated receptor
SphK2	sphingosine kinase 2
SREBP	sterol regulatory element binding protein
