# Supplementary material for: Current Challenges in Understanding the Cellular and Molecular Mechanisms in Niemann–Pick Disease Type C1
Source: Int J Mol Sci. 2019 Sep 6;20(18):4392. doi: 10.3390/ijms20184392 (PMC6771135; doi:10.3390/ijms20184392)
Supplement: Supplementary file 1 [file ijms-20-04392-s001.pdf]

International Journal of *Molecular Sciences*

Supplementary Methods

1.1. Animals

Heterozygous breeding pairs of NPC1 mice (BALB/cNctr-*Npc1*$^{m1N/-}$J) were obtained from Jackson Laboratories (Bar Harbor, ME, USA) for generating homozygous *Npc1*$^{-/-}$ mutants and *Npc1*$^{+/+}$ control wild type mice. Mice were maintained under standard conditions with free access to food and water with a 12 h day/night cycle, a temperature of 22 °C and a relative humidity of 60%. Genotypes were determined until postnatal day P7 by PCR analysis.

All animal procedures were approved by the local State Animal Research Committee of Mecklenburg-Western Pomerania (approval ID: 7221.3-1.1-011/16).

1.2. Sample Preparation and Histology

Mice were deeply anesthetized with a mixture of 50 mg/kg ketamine hydrochloride (Bela-Pharm GmbH & Co KG, Vechta, Germany) and 2 mg/kg body weight of xylazine hydrochloride (Rompun; Bayer HealthCare, Leverkusen, Germany) and then intracardially perfused with normal saline solution, followed by 4% paraformaldehyde (PFA) in 0.1 M PBS. Mice were then decapitated, several organs, including the whole brain, dissected and post-fixed in 4 % PFA for 24h at 4 °C. Subsequently, specimens were dehydrated and embedded in paraffin. Tissues were cut in 5–10 µm thick sections and routine stained with hematoxylin and eosin.

1.3. Electron microscopy

After initial perfusion and preparation (see above), samples of five *Npc1*$^{-/-}$ and *Npc1*$^{+/+}$ mice were postfixed in 0.1 M cacodylate buffer containing 2.5% glutaraldehyde for at least 24 hours at 4 °C. Subsequently, small (1 mm^3) samples of various organs were excised and kept in the same fixative. Thereafter, the specimens were osmicated, washed, block contrasted with 2% aqueous uranyl acetate, dehydrated through a graded series of ethanol, and embedded in Epon 812 (Plano GmbH, Marburg, Germany). Ultrathin sections (about 70 nm) were mounted on pioloform-coated slot copper grids and contrasted with uranyl acetate (4 minutes) followed by lead citrate (2 minutes). The specimens were examined with a Zeiss EM 902 transmission electron microscope (Zeiss, Oberkochen, Germany) at 80 kV. Photographs were taken using a CCD camera (Proscan, Lagerlechfeld, Germany) and adjusted using Photoshop CS2 software (Adobe Systems, San Jose, CA, USA).
